# Supplementary material for: Herpes Simplex Virus Mistyping due to HSV-1 × HSV-2 Interspecies Recombination in Viral Gene Encoding Glycoprotein B
Source: Viruses. 2020 Aug 6;12(8):860. doi: 10.3390/v12080860 (PMC7472045; doi:10.3390/v12080860)
Supplement: Supplementary file 1 [file viruses-12-00860-s001.pdf]

**Supplementary Methods**

Primer Sequences for Thymidine Kinase (UL23) and DNA Polymerase (UL30) Amplification

Thymidine Kinase

- HSV-1
  - F AACACCCGTGCGTTTTATTC
  - R ATATTAAGGTGACGCGTGTGG
- HSV-2
  - F ACACACCACACGACAACAATG
  - R CGAGGTCCACTTCGCATATT

DNA Polymerase

- HSV-1
  - Set 1
    - F TTTAACTGTACGGCGGACAAC
    - R GTGAGCCTTGACGAAGAACAG
  - Set 2
    - F ATTAACATCACCCGCACCAT
    - R TCTTACCCCCGTAGATGACG
  - Set 3
    - F CACGGACTCCATATTTGTGCT
    - R TCTATGCAACATTCGACGAGTT
- HSV-2
  - Set 1
    - F GCACGCGGCAGTACTTTTAC
    - R AAGTTATCGCACAGGTAGGC
  - Set 2
    - F GCGAGTATTGTGTGCAGGACT
    - R ATAAACGCGCAGTTGTTTTTG

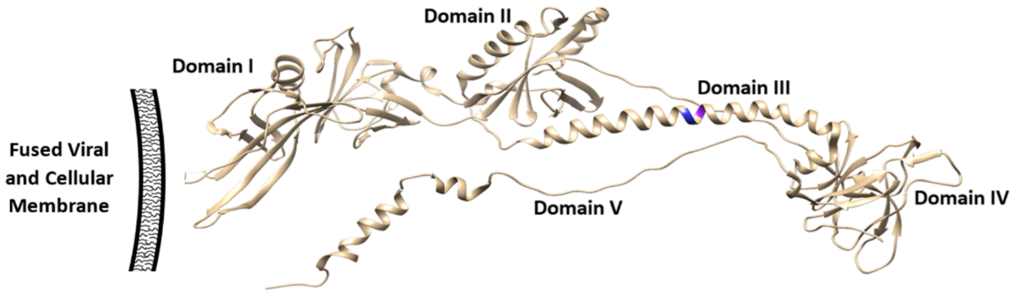


**Supplementary Figure S1.** Postfusion protein conformation of glycoprotein B of HSV-1. Residue 527 (V) is shown in blue and residue 529 (I) is shown in purple. The protein is divided in five structural domains. Residues 527 and 529 are within Domain III of glycoprotein B, which is thought to be important in the binding of three glycoprotein peptides together to form the active trimer. The location of the fused viral and cellular membrane relative to the different domains of the postfusion protein is indicated.

| List of Genes in HSV with Known HSV-1 x HSV-2 Recombination Events | |
|---|---|
| **Gene** | **Protein Product** |
| UL15 | DNA packaging terminase subunit 1 |
| UL17 | DNA packaging tegument protein |
| UL27 | Glycoprotein B |
| UL28 | DNA packaging terminase subunit 2 |
| UL29 | Single-stranded DNA-binding protein |
| UL30 | DNA polymerase |
| UL31 | Nuclear egress lamina protein |
| UL32 | DNA packaging protein |
| UL39 | Ribonucleotide reductase large subunit |
| UL47 | Tegument protein VP13/14 |
| UL48 | Transactivating tegument protein VP16 |
| UL49 | Tegument protein VP22 |
| UL49A | Glycoprotein N |
| UL50 | Deoxyuridine triphosphatase |

**Supplementary Table S1:** HSV genes with known HSV-1 x HSV-2 recombination events.
